# Body Weight Selection Affects Quantitative Genetic Correlated Responses in Gut Microbiota

**DOI:** 10.1371/journal.pone.0089862

**Published:** 2014-03-07

**Authors:** He Meng, Yan Zhang, Lele Zhao, Wenjing Zhao, Chuan He, Christa F. Honaker, Zhengxiao Zhai, Zikui Sun, Paul B. Siegel

**Affiliations:** 1 Shanghai Key Laboratory of Veterinary Biotechnology, School of Agriculture and Biology, Shanghai Jiao Tong University, Shanghai, P. R. China; 2 School of Agriculture and Biology, Shanghai Jiao Tong University, Shanghai, P. R. China; 3 Virginia Bioinformatics Institute, Virginia Polytechnic Institute and State University, Blacksburg, Virginia, United States of America; 4 Department of Animal and Poultry Sciences, Virginia Polytechnic Institute and State University, Blacksburg, Virginia, United States of America; 5 Shanghai Personal Biotechnology Limited Company, Shanghai, P. R. China; University of Queensland, Australia

## Abstract

The abundance of gut microbiota can be viewed as a quantitative trait, which is affected by the genetics and environment of the host. To quantify the effects of host genetics, we calculated the heritability of abundance of specific microorganisms and genetic correlations among them in the gut microbiota of two lines of chickens maintained under the same husbandry and dietary regimes. The lines, which originated from a common founder population, had undergone >50 generations of selection for high (HW) or low (LW) 56-day body weight and now differ by more than 10-fold in body weight at selection age. We identified families of *Paenibacillaceae*, *Streptococcaceae*, *Helicobacteraceae*, and *Burkholderiaceae* that had moderate heritabilities. Although there were no obvious phenotypic correlations among gut microbiota, significant genetic correlations were observed. Moreover, the effects were modified by genetic selection for body weight, which altered the quantitative genetic background of the host. Heritabilities for *Bacillaceae*, *Flavobacteriaceae*, *Helicobacteraceae*, *Comamonadaceae*, *Enterococcaceae*, and *Streptococcaceae* were moderate in LW line and little to zero in the HW line. These results suggest that loci associated with these microbiota families, while exhibiting genetic variation in LW, have been fixed in HW line. Also, long term selection for body weight has altered the genetic correlations among gut microbiota. No microbiota families had significant heritabilities in both the LW and HW lines suggesting that the presence and/or absence of a particular microbiota family either has a strong growth promoting or inhibiting effect, but not both. These results demonstrate that the quantitative genetics of the host have considerable influence on the gut microbiota.

## Introduction

High levels of diversity, community structure, and composition of gut microbiota are strongly associated with host species and are very stable and consistent within host species [1], [2], reflecting selection of microorganisms by their host and environmental factors. Within host species, factors such as diet, litter, and maternal effects [3]–[6], as well as single gene differences, affect the population structure of gut microbiota [7]–[10]. Previously, we reported that the quantitative genotype of the host influenced gut microbiota composition in adults from two lines of chickens fed a common diet and maintained under the same husbandry [11]. This finding suggests that the gut microbiome can be viewed as a complex, polygenic trait with a synergy of host genetic and environmental factors that shape and account for variability. Until now, the role of host genetics on shaping this vital ‘microbial organ’ was not clear, and to our knowledge, lacking quantification of contributions from the polygenic background of the host.

**Figure 1:**
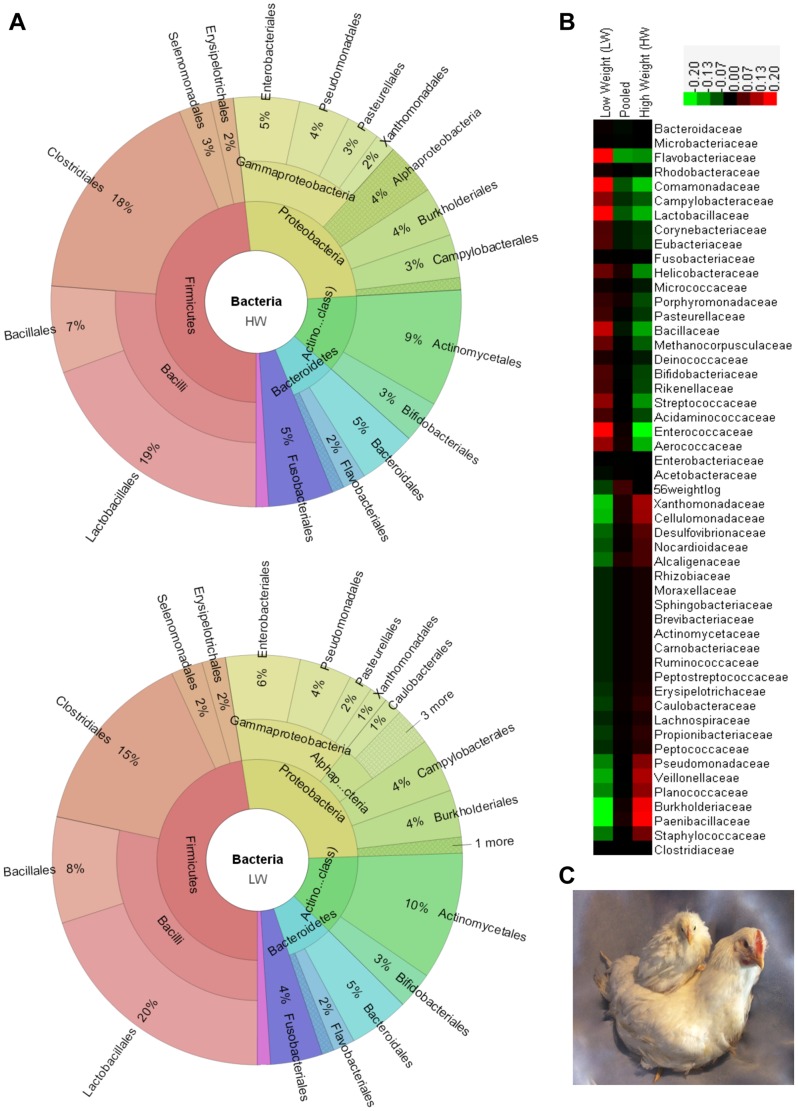
High (HW) and low (LW) lines of chicken. A. The distribution of the gut microbiomes composite for HW and LW lines. B. Some heritabilities of taxonomy in these lines. C. Picture of high (HW) and low (LW) line females.

The genotype of the host may affect its microbiota composition directly through secretions into the gut, influences on gut motility and modification of epithelial cell surfaces, or indirectly through food and lifestyle preferences. Detecting their role(s) will require well controlled effects other than those of the host genotype. Thus, choosing a model organism maintained in essentially an identical environment with few maternal effects should enhance our understanding of host genotype effects on gut microbiota. Here, we choose for our model two lines of chickens that had undergone >50 generations of bidirectional selection for 56-day high (HW) or low (LW) body weight (Figure 1). The lines originated from a common founder population and have complete pedigrees [12], [13]. Throughout all generations, they have been maintained at the same location and reared on the same diets. Selection has resulted in more than a 10-fold difference between them for body weight at selection age. They are segregating populations with maximum inbreeding coefficients (F) being 0.53 and 0.61, with a mean of 0.26 (SD 0.15) and 0.30 (SD 0.17) in LW and HW lines, respectively [13]. QTL mapping revealed 13 loci affecting growth in these two lines, however, each locus explained only a small additive effect for this large phenotypic difference [14]. This moderate F, along with QTL results, suggest that there is genetic diversity within each line, and the magnitude of the body weight difference between them at selection age is because of an accumulation of quantitative genes, each with very small effects rather than a single gene mutation. Not only do these lines provide an ideal model for studying quantitative genetic effects of the host on the microbiome of the gut, they also help us characterize how selection pressures on the host alter its genetic impact on the gut microbiota. Aided by next generation sequencing technology, we investigated the population structure of the gut microbiota in adults of these two lines. We treated the abundance of microorganisms of gut microbiota as a quantitative trait of the host by calculating the heritabilities of abundance of specific microorganisms and quantifying diversity and composition of community structure of the gut microbiome based on the host's quantitative genetic background. Genetic correlations were calculated among different microorganisms as they allow for quantifying the contribution from the host's genetic background to the interaction among the different microorganisms.

## Results and Discussion

At least 22,000 sequence reads, which passed our quality criteria, were generated for each sample. The average sequence reads per sample was 42,358. After taxonomic classification, 50 families, which were present in at least 65 samples, were considered as common and their normalized abundance counts [11] were used for further analysis.

### Selection pressure alters additive effects from the host's genetic background for the gut microbiome

The abundance of microorganisms of gut microbiota, as a quantitative trait of the host, is affected by the additive effects from host genotype + environmental factors + random error. The heritability of a quantitative trait estimates the fraction of the phenotypic variation attributed to genetic variation. Here, we calculated the heritability of abundance of each microorganism to estimate the additive effects of the host's quantitative genetic background (Table 1). The heritability of 56-day body weight for the pooled group (combine the two lines and treat line and sex as fixed effects) was 0.326. This value was consistent with estimates for this trait in other populations, and suggests that the moderate heritability of body weight for these populations is typical for that in chickens. Four families of microbiota (*Paenibacillaceae*, *Streptococcaceae*, *Helicobacteraceae*, and *Burkholderiaceae*) had moderate heritabilities (Table 1).

**Table:** **Table 1.** Heritabilities by line and pooled.

	Pooled	Low Weight	High Weight
**56-day body weight**	**0.326** *	0.263	0.265
***Fusobacteriaceae***	0.013	0.066	0.000
***Deinococcaceae***	0.211	0.280	0.179
***Erysipelotrichaceae***	0.029	0.018	0.032
***Ruminococcaceae***	0.000	0.000	0.000
***Peptococcaceae***	0.023	0.017	0.033
***Eubacteriaceae***	0.045	0.174	0.011
***Peptostreptococcaceae***	0.000	0.000	0.000
***Lachnospiraceae***	0.000	0.000	0.004
***Clostridiaceae***	0.006	0.044	0.000
***Acidaminococcaceae***	0.106	0.202	0.034
***Veillonellaceae***	0.090	0.000	0.214
***Paenibacillaceae***	**0.534** **	0.238	**0.787** **
***Planococcaceae***	0.058	0.000	0.164
***Bacillaceae***	0.195	**0.409** *	0.077
***Staphylococcaceae***	0.075	0.017	0.151
***Carnobacteriaceae***	0.000	0.000	0.000
***Aerococcaceae***	0.170	0.310	0.000
***Enterococcaceae***	0.245	**0.473** **	0.000
***Lactobacillaceae***	0.079	0.402	0.000
***Streptococcaceae***	**0.210** *	**0.360** *	0.080
***Bifidobacteriaceae***	0.122	0.233	0.062
***Nocardioidaceae***	0.099	0.063	0.138
***Propionibacteriaceae***	0.201	0.183	0.216
***Actinomycetaceae***	0.000	0.000	0.000
***Corynebacteriaceae***	0.031	0.158	0.000
***Microbacteriaceae***	0.003	0.059	0.000
***Brevibacteriaceae***	0.000	0.000	0.000
***Cellulomonadaceae***	0.128	0.000	0.214
***Micrococcaceae***	0.115	0.167	0.076
***Sphingobacteriaceae***	0.000	0.000	0.000
***Flavobacteriaceae***	0.000	**0.400** *	0.000
***Porphyromonadaceae***	0.091	0.155	0.000
***Rikenellaceae***	0.152	0.248	0.080
***Bacteroidaceae***	0.000	0.066	0.000
***Desulfovibrionaceae***	0.127	0.075	0.175
***Campylobacteraceae***	0.046	0.234	0.000
***Helicobacteraceae***	**0.331** **	**0.429** **	0.185
***Burkholderiaceae***	**0.454** **	0.277	**0.613** **
***Comamonadaceae***	0.198	**0.530** *	0.100
***Alcaligenaceae***	0.085	0.005	0.101
***Xanthomonadaceae***	0.140	0.000	0.227
***Pasteurellaceae***	0.065	0.166	0.016
***Enterobacteriaceae***	0.000	0.048	0.000
***Pseudomonadaceae***	0.060	0.000	0.154
***Moraxellaceae***	0.000	0.000	0.000
***Rhizobiaceae***	0.000	0.000	0.000
***Caulobacteraceae***	0.038	0.015	0.053
***Rhodobacteraceae***	0.112	0.177	0.092
***Acetobacteraceae***	0.196	0.216	0.181
***Methanocorpusculaceae***	0.080	0.210	0.000

*: p<0.05,

**: p<0.01.

Although the two lines of chickens reported here originated from the same founder population, after >50 generations of long term pedigree selection there were changes in gene frequencies that could have altered their quantitative genetic background [14]. Their phenotypes (Figure 1) and physiological functions are quite different [13], [15]. To determine if the changing quantitative genetic background of the host affected the additive effects for gut microbiota, we calculated heritabilities within each line, and the results were quite different (Table 1). Seventeen families had heritabilities greater than 0.20 in the LW line, 6 in the HW line, with the overlap between lines of only 2 families. Heritabilities of *Bacillaceae*, *Flavobacteriaceae*, *Helicobacteraceae*, *Comamonadaceae*, *Enterococcaceae*, and *Streptococcaceae* were moderate (p<0.05) in the LW line and little to zero in HW line. Families of *Aerococcaceae*, *Enterococcaceae*, *Lactobacillaceae*, and *Streptococcaceae* belong to Lactobacillales, the order of most probiotics. Their heritabilities were moderate in LW and little to zero in HW. After >50 generations of selection for high or low body weight under a common diet, one may speculate that the quantitative genetic background is quite different in the two lines and genes which enhance growth in line HW have afforded sufficient absorption of nutrients so that over time there was less selection pressure for gut microbiota in HW. This suggests that for the LW line, there was greater need for these microorganisms, hence, greater effects from the host. This reasoning also supports the thesis that abundance of some microorganisms is determined by the genetic background of the host with the effect of each gene being small.

### Genetic correlations to quantify the association of host genetic background to microorganism interactions

The genetic correlation is the proportion of variance (i.e. covariance) that two traits share due to common genetics. That the heritability of some microorganisms is moderate implies that they are influenced by the genotype of the host. Accordingly, we calculated genetic and phenotypic correlations among microorganisms in the gut microbiome whose heritabilities were greater than 0.20 (Table 2). Although there were no obvious phenotypic correlations among gut microorganisms, some genetic correlations were high, such as *Streptococcaceae* and *Propionibacteriaceae* (0.904**) and *Paenibacillaceae* and *Bacillaceae* (0.804**), while others such as *Streptococcaceae* and *Comamonadaceae* (−0.524**) and *Helicobacteraceae* and *Comamonadaceae* (0.480**) were moderate. We also calculated phenotypic and genetic correlations among gut microorganisms in the HW (Table S1) and LW (Table S2) lines, respectively. The phenotypic correlations between *Burkholderiaceae* and *Paenibacillaceae* were small in both lines (Table S2, Table S3), however, the genetic correlations were moderate (−0.442) in LW and high (−0.711**) in HW chickens. These results imply further the importance of the genetic background of the host on the interactions among microbiota.

**Table 2:** Genetic and phenotypic correlations for lines pooled.

	56-day body weight	*Deinococcaceae*	*Propionibacteriaceae*	*Paenibacillaceae*	*Bacillaceae*	*Enterococcaceae*	*Streptococcaceae*	*Burkholderiaceae*	*Comamonadaceae*	*Helicobacteraceae*	*Acetobacteraceae*
**56-day body weight**	**0.326** *	0.061	−0.040	−0.113	−0.086	−0.198	−0.087	−0.093	−0.023	0.169	−0.042
***Deinococcaceae***	0.513*	**0.211**	0.261	−0.213	−0.191	0.030	0.319	0.073	−0.110	−0.164	−0.236
***Propionibacteriaceae***	0.237	−0.006	**0.201**	−0.227	−0.233	0.183	0.516	0.037	−0.186	−0.367	−0.267
***Paenibacillaceae***	−0.449*	−0.358	−0.241	**0.534** **	0.545	0.166	−0.151	−0.013	0.274	0.095	0.021
***Bacillaceae***	−0.433*	−0.199	−0.418*	0.804**	**0.195**	0.235	−0.207	0.131	0.058	−0.016	0.100
***Enterococcaceae***	−0.627**	−0.488*	0.392	−0.274	−0.495*	**0.245**	0.475	−0.079	0.092	−0.588	0.003
***Streptococcaceae***	−0.141	−0.015	0.904**	−0.445*	−0.720**	0.580*	**0.210** *	0.071	−0.116	−0.666	−0.162
***Burkholderiaceae***	0.316	0.129	0.289	−0.536**	−0.411*	−0.690**	0.113	**0.454** **	−0.100	−0.085	0.085
***Comamonadaceae***	−0.140	0.000	−0.486*	0.623**	0.345	0.242	−0.524*	−0.498*	**0.198**	−0.024	0.093
***Helicobacteraceae***	0.167	0.257	−0.458*	0.429*	0.283	−0.579*	−0.773**	−0.045	0.480*	**0.331** **	0.033
***Acetobacteraceae***	−0.524*	0.460*	−0.282	−0.510*	−0.065	0.430*	0.403	0.369	0.084	−0.551*	**0.196**

upper side triangle are phenotypic correlations, lower side triangle are genetic correlations with heritabilities on the diagonal.

*: p<0.05,

**: P<0.01.

### Correlations of 56-day body weight per se with gut microbiota

Because 56-day body weight is a quantitative trait, genetic and phenotypic correlations between it and microorganisms whose heritability was greater than 0.20 were calculated (Table 2). Although no phenotypic correlation between 56-day body weight and microorganisms was significant, the genetic correlation with *Deinococcaceae* (0.513*) was positive while those for *Paenibacillaceae* (−0.449*), *Bacillaceae* (−0.433*), *Enterococcaceae* (−0.627**) and *Acetobacteraceae* (−0.524*) were negative (Table 2). To determine if these relationships were consistent after long term selection, we also calculated the phenotypic and genetic correlations between 56-day body weight and gut microorganism in the HW (Table S1) and LW (Table S2) lines, respectively. Both *Burkholderiaceae* and *Paenibacillaceae* had moderate heritabilities in both lines, however, their interaction with 56-day body weight was reversed. For *Burkholderiaceae*, the genetic correlation was −0.722 in HW, and 0.583 in LW. For *Paenibacillaceae*, the genetic correlation was 0.543 in HW and −0.747 in LW, implying further that no major single gene was responsible for host-microorganism interactions. Rather, they are influenced by several genes each with small effects and can be bidirectionally dependent on the host.

### General 0043omments

Heritabilities of *Bacillaceae*, *Flavobacteriaceae*, *Helicobacteraceae*, *Comamonadaceae*, *Enterococcaceae*, and *Streptococcaceae* were moderate (p<0.05) in the LW line and little to zero in the HW line. These results suggest that genes which contribute to the abundance of these microorganisms families may be fixed in the HW but not in the LW line. It also can be deduced that the presence and/or absence of these microbe families are more important in the HW line because the heritability is 0 implying no genetic variation. Because the heritability of these families are greater than 0 in one of the lines (LW) then genetic variation contributing to these microbiota families was likely present in the founding population. There is a novel deletion that is fixed in the HW line and occurs at a low frequency in the LW line, which contributes to the differences in growth [16] and feed efficiency [Siegel, unpublished]. Combined with our results, it suggests that some genes located in the deletion region may be associated with microbiota families.

None of the microbiota families that we measured had significant heritabilities in both the LW and HW lines, suggesting that the presence and/or absence of a particular microbiota family can either have a strong growth promoting or inhibiting effect, but not both.

In order to avoid reproduction problems and metabolic issues caused by obesity, the HW line chickens used in this experiment were feed restricted starting at 56 days, which was selection age. This practice initiated in generation S18, was necessary to prevent obesity from overconsumption from ad libitum feeding. Because the objective of this experiment was to address if the genetic background of the host influenced the gut microbiome community, we collected fecal samples at 245 days of age when the host was mature. Studies show that dietary composition and caloric intake appear to swiftly regulate intestinal microbial composition and function [17], [18]. Therefore, fecal samples were taken 189 days after feed restriction was started, a period that should have allowed the chickens to sufficiently adjust to issues associated with feed restriction.

Our results are consistent with the thesis that the genetic background of the host influences the diversity and composition of gut microbiome. Involved are numerous genes, each having a small additive effect. Moreover, the quantitative genetic expression of the host will change over time due to selection, resulting in different families of microorganisms. Although gut microbiota could influence physiological functions of the host, and vice versa, its accumulation in the gut is greatly influenced by the host's genotype.

## Conclusions

Families of *Paenibacillaceae*, *Streptococcaceae*, *Helicobacteraceae*, and *Burkholderiaceae* had moderate heritabilities, which provide evidence that the abundance of gut microbiota can be viewed as a quantitative trait and evidence of an association between host genome and gut microbiota. Moreover, these associations have been modified by genetic selection for body weight, which altered gene frequencies of the host resulting in a change in its quantitative genetic background. The outcome of the divergent selection is that heritabilities for *Bacillaceae*, *Flavobacteriaceae*, *Helicobacteraceae*, *Comamonadaceae*, *Enterococcaceae*, and *Streptococcaceae* were moderate and little to zero in the LW and HW lines, respectively.

Although there were no obvious phenotypic correlations among gut microbiota, there were significant genetic correlations, implying that the long term selection for body weight also altered the genetic correlations among gut microbiota. Therefore some of interactions among gut microbiota are mediated by the host. These mediation are through host genes, and changing these gene frequencies will also modify interactions among gut microbiota. Thus, the quantitative genetic background of the host can have considerable influence on the gut microbiota.

## Materials and Methods

### Animals and sample collection

Protocols used for this experiment were approved by the Institutional Animal Care and Use Committee at Virginia Tech. Fresh fecal samples from 258 adult chickens, which consisted of 88 LW females, 38 LW males, 90 HW females, and 42 HW males, were collected. The fecal samples were obtained from generations 54 and 55 of the HW and LW lines when the chickens were 245 days of age. These chickens had been under similar husbandry conditions and were fed a corn-soybean, non-pathogen free diet in mash form. Details are provided in the supplement of breeder diet ingredients. Cages had sloping wire floors with papers beneath them to collect feces.

### DNA extraction, PCR amplification of 16S rRNA, amplicon sequence and sequence data processing

Microbial genome DNA was extracted from fecal samples using QIAamp DNA stool mini kit (QIAGEN, cat#51504) following the manufacturer's recommendation. According to our previous comparisons, results from V3, V4, V1–V3, and V4–V6 hypervariable regions of 16S rRNA [11], V4 of 16S rRNA were PCR amplified from microbial genome DNA harvested from fecal samples using barcoded fusion primers (forward primers: 5′AYTGGGYDTAAAGNG 3′, reverse primers: 5′TACNVGGGTATCTAATCC 3′) and were used for the remainder of our study. The PCR condition and PCR product purification followed our previous publication [11]. Barcoded V4 amplicons were sequenced using the pair-end method by Illumina Miseq with a 6 cycle index read. Sequences with an average phred score lower than 25, containing ambiguous bases, homopolymer run exceeds 6, having mismatches in primers, or sequence length shorter than 100 bp were removed. Only sequences with an overlap longer than 10 bp and without any mismatch were assembled according to their overlap sequence. Reads which could not be assembled were discarded. Barcode and sequencing primers were trimmed from assembled sequence. Trimmed sequences were uploaded to MGRAST [19] for further study.

### Taxonomy classification and statistical analysis

The taxon abundance of each sample was generated into family levels primarily using the RDP database, aided by Greengene, and SSU databases. Each sample's trimmed sequences were compared to the RDP, Greengene, and SSU databases using the best hit classification option to classify the abundance count of each taxon. The classification parameters were 8 for maximum e-value cutoff, 98 for minimum % identity cutoff, and 120 bp for minimum alignment length cutoff. This process was archived by MGRAST [19]. The metagenome sequences used in this paper are publicly available from the MGRAST (http://metagenomics.anl.gov/).

The abundance count for family level was transformed by log2, and then normalized per our previous report [11]. After this procedure, the abundance profiles for all samples will exhibit a mean of 0 and a standard deviation of 1. Heritabilities, genetic correlations, and estimations of their accuracies were calculated using AI-REML algorithm by DMU (http://www.dmu.agrsci.dk/dmuv6_guide-R4-6-7.pdf).

## Supporting Information

File S1 Descriptions of the HW and LW lines of chickens and their diet ingredients.
(DOC)

Table S1 Genetic and phenotype correlations for line HW.
(DOCX)

Table S2 Genetic and phenotype correlations for line LW.
(DOCX)

Table S3 Body weight and egg production of HW and LW lines of chicken.
(DOCX)
