# Correction: Body Weight Selection Affects Quantitative Genetic Correlated Responses in Gut Microbiota

**DOI:** 10.1371/journal.pone.0101401

**Published:** 2014-06-20

**Authors:** The PLOS ONE Staff

There are errors in the author affiliations. The affiliations should appear as shown here:

He Meng^1*‡^, Yan Zhang^2*‡^, Lele Zhao^1^, Wenjing Zhao^1^, Chuan He^4^, Christa F. Honaker^3^, Zhengxiao Zhai^1^, Zikui Sun^4^, Paul B. Siegel^3*^

* Corresponding authors

‡ These authors contributed equally to this work

1. School of Agriculture and Biology, Shanghai Jiaotong University; Shanghai Key Laboratory of Veterinary Biotechnology, Shanghai, P. R. China.

2. Virginia Bioinformatics Institute, Virginia Tech, Blacksburg, Virginia, USA.

3. Department of Animal and Poultry Sciences, Virginia Tech, Blacksburg, Virginia, USA.

4. Shanghai Personal Biotechnology Limited Company, Shanghai, P.R. China.

Correspondence and requests for materials should be addressed to: He Meng: menghe@sjtu.edu.cn, Yan Zhang: yzhang@vbi.vt.edu, Paul Siegel: pbsiegel@vt.edu
