# Supplementary material for: Body Weight Selection Affects Quantitative Genetic Correlated Responses in Gut Microbiota
Source: PLoS One. 2014 Mar 7;9(3):e89862. doi: 10.1371/journal.pone.0089862 (PMC3946484; doi:10.1371/journal.pone.0089862)
Supplement: File S1 — Descriptions of the HW and LW lines of chickens and their diet ingredients. (DOC) [file pone.0089862.s001.doc]

**Supplemental material**

**Body weight selection affects quantitative genetic correlated responses in gut microbiota**

He Meng^1,*,‡^, Yan Zhang^2,*,‡^, Lele Zhao^1^, Wenjing Zhao^1^, Chuan He^4^, Christa F. Honaker^3^, Zhengxiao Zhai^1^, Zikui Sun^4^, Paul B. [Siegel](http://www.apsc.vt.edu/people/biographies/faculty/siegel/siegel-bio.html)^3, *^

* Corresponding authors

‡ These authors contributed equally to this work

1. School of Agriculture and Biology, Shanghai Jiaotong University; Shanghai Key Laboratory of Veterinary Biotechnology, 800 Dongchuan Road, Shanghai 200240, P. R. China.

2. Virginia Bioinformatics Institute, Virginia Tech, Washington Street, MC0477, Blacksburg, Virginia, 24061, USA.

3. Department of Animal and Poultry Sciences, Virginia Tech, Blacksburg, Virginia, 24061, USA

4. Shanghai Personal Biotechnology Limited Company, 777 Longwu Road, Shanghai 200336, P.R. China.

(He Meng: [menghe@sjtu.edu.cn](mailto:menghe@sjtu.edu.cn), Yan Zhang: [yzhang@vbi.vt.edu](mailto:yzhang@vbi.vt.edu), Paul B. Siegel: [pbsiegel@vt.edu](mailto:pbsiegel@vt.edu))

**Descriptions of the high and low body weight lines of chickens**

Body weight, at a particular point in time, is a complex trait with both genetic and nongenetic components. A long-time selection experiment for high (HW) and low (LW) body weight at 56 days of age is now in its 55^th^ generation. The founder population consisted of a cross of 7 moderately inbred lines of White Plymouth Rock chickens. From the initial generation each line was reproduced annually in March and pedigrees have been maintained.

Response to selection was immediate and the difference between the lines in body weight at selection age was more than 10-fold in generation 55 (Supplementary Table S4). Concomitant to changes in body weight were numerous correlated responses with feed utilization being significantly superior in the high than low line as early as generation S5. Earlier, Wisman and Siegel reported that by generation S3 the lines differed in their protein and energy requirements. Since then, these lines have been a source for numerous studies on growth, feed utilization and food intake. During the course of selection there has been a conscious effort to minimize environmental factors. Dietary formulations have remained the same with the only feed additive being a coccidiostat in the starter and developer. The diets are corn-soybean based and are provided in mash form. From hatch to selection age, the starter diet consists of 20% crude protein and 2,685 kcalME/kg. From day 57 to approximately day 140 the developer diet feed consists of 16% crude protein and 2, 761 kcal ME/kg. Thereafter, the chickens are fed a 16.1% crude protein and 2,752 kcal ME/kg breeder diet. (The chickens used in this experiment were placed on the breeder diet at 140 days of age). Until generation 18, feed was provided *ad libitum*. Then a feed restriction program was instituted for the high weight line to reduce reproductive issues associated with obesity.

# The breeder diet ingredients ground yellow corn, pulverized oats, wheat flower middling, stabilized fat, dehulled soybean meal, fish meal, meat and bone scraps, deflorinated phosphate, ground limestone, iodized salt, trace mineral mix [manganese, zinc, copper, iodine, cobalt, calcium] vitamin premix [vitamins A, B12, D3, and E, menadine sodium bisulfate, riboflavin, calcium d-pantothenate, niacin, choline chloride, folic acid, DL-methionine, selenium, ethoxyquin]

# Reference

# 1. Dunnington EA, Siegel PB (1996) Long-term divergent selection for eight-week body weight in white Plymouth rock chickens. Poult Sci 75: 1168-1179.

# 2. Marquez GC, Siegel PB, Lewis RM (2010) Genetic diversity and population structure in lines of chickens divergently selected for high and low 8-week body weight. Poult Sci 89: 2580-2588.

# 3. Ideta G, Siegel PB (1966) Selection for body weight at eight weeks of age. 4. Phenotypic, genetic and environmental correlations between selected and unselected traits. Poult Sci 45: 939-945.

# 4. Siegel ELWaPB (1963) Further studies on protein and energy requirements of chicks selected for high and low body weight. . poultry science 42: 541-543.

# 5. O'Sullivan NP, Dunnington EA, Siegel PB (1992) Correlated responses in lines of chickens divergently selected for fifty-six-day body weight. 1. Growth, feed intake, and feed utilization. Poult Sci 71: 590-597.

# 6. Cline MA, Nandar W, Bowden C, Calchary W, Smith ML, et al. (2010) The threshold of amylin-induced anorexia is lower in chicks selected for low compared to high juvenile body weight. Behav Brain Res 208: 650-654.
