# Supplementary material for: Body Weight Selection Affects Quantitative Genetic Correlated Responses in Gut Microbiota
Source: PLoS One. 2014 Mar 7;9(3):e89862. doi: 10.1371/journal.pone.0089862 (PMC3946484; doi:10.1371/journal.pone.0089862)

**Table S1: Genetic and phenotype correlations for line HW**

#: upper side triangle are phenotypic correlations, lower side triangle are genetic correlations with heritabilities on the diagonal. *: p<0.05, **: P<0.01

|  | **56-day body weight** | ***Burkholderiaceae*** | ***Xanthomonadaceae*** | ***Paenibacillaceae*** | ***Veillonellaceae*** | ***Cellulomonadaceae*** | ***Propionibacteriaceae*** |
| --- | --- | --- | --- | --- | --- | --- | --- |
| **56-day body weight** | **0.265** | -0.150 | 0.071 | 0.044 | -0.034 | -0.007 | 0.050 |
| ***Burkholderiaceae*** | -0.722** | **0.613**** | -0.070 | -0.204 | -0.097 | -0.265 | 0.106 |
| ***Xanthomonadaceae*** | 0.761** | -0.590* | **0.227** | 0.332 | -0.045 | 0.330 | 0.000 |
| ***Paenibacillaceae*** | 0.543* | -0.711** | 0.555* | **0.787**** | -0.170 | 0.242 | 0.025 |
| ***Veillonellaceae*** | -0.568* | -0.119 | -0.450 | -0.383 | **0.214** | -0.211 | -0.031 |
| ***Cellulomonadaceae*** | 0.166 | -0.543* | 0.610** | 0.738** | -0.279 | **0.214** | -0.144 |
| ***Propionibacteriaceae*** | -0.815** | 0.072 | 0.000 | 0.001 | -0.435 | -0.775** | **0.216** |


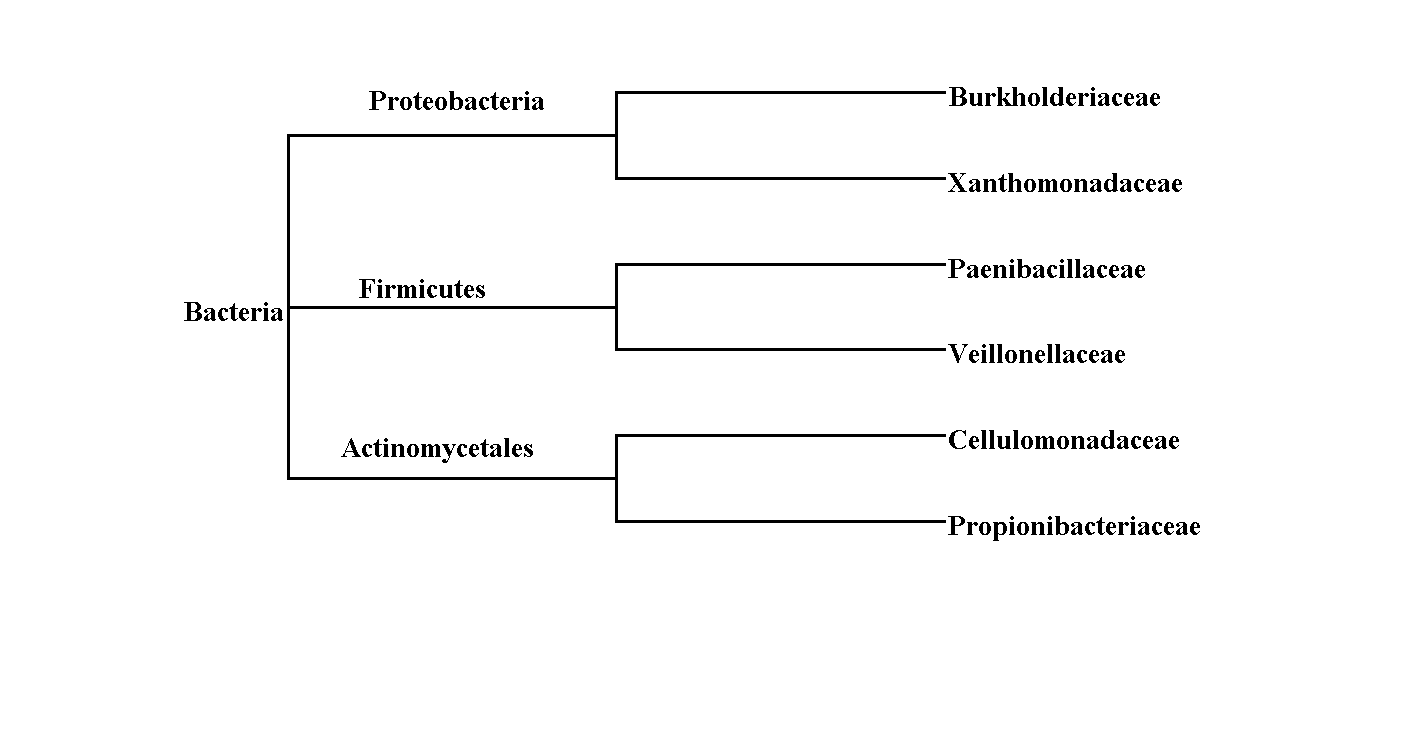

Supplement: Table S1 — Genetic and phenotype correlations for line HW. (DOCX) [file pone.0089862.s002.docx]
