# Supplementary material for: Body Weight Selection Affects Quantitative Genetic Correlated Responses in Gut Microbiota
Source: PLoS One. 2014 Mar 7;9(3):e89862. doi: 10.1371/journal.pone.0089862 (PMC3946484; doi:10.1371/journal.pone.0089862)

**Table S2 Genetic and phenotypic correlations for line LW**

#: upper side triangle are phenotypic correlations, lower side triangle are genetic correlations with heritabilities on the diagonal. *: p<0.05, **: P<0.01

|  | **56-day body weight** | ***Methanocorpusculaceae*** | ***Burkholderiaceae*** | ***Comamonadaceae*** | ***Campylobacteraceae*** | ***Helicobacteraceae*** | ***Acetobacteraceae*** | ***Bifidobacteriaceae*** | ***Rikenellaceae*** | ***Flavobacteriaceae*** | ***Deinococcaceae*** | ***Acidaminococcaceae*** | ***Paenibacillaceae*** | ***Bacillaceae*** | ***Aerococcaceae*** | ***Enterococcaceae*** | ***Lactobacillaceae*** | ***Streptococcaceae*** |
| --- | --- | --- | --- | --- | --- | --- | --- | --- | --- | --- | --- | --- | --- | --- | --- | --- | --- | --- |
| **56-day body weight** | **0.263** | -0.034 | -0.103 | -0.003 | -0.253 | 0.288 | -0.038 | 0.000 | 0.215 | 0.058 | 0.030 | -0.018 | -0.136 | -0.167 | -0.219 | -0.306 | 0.141 | -0.002 |
| ***Methanocorpusculaceae*** | -0.185 | **0.210** | -0.127 | -0.062 | -0.236 | 0.218 | -0.066 | -0.052 | 0.239 | -0.213 | 0.000 | 0.044 | -0.016 | 0.002 | -0.224 | -0.125 | 0.032 | -0.319 |
| ***Burkholderiaceae*** | 0.583 | -0.496 | **0.277** | -0.136 | 0.017 | -0.147 | 0.102 | -0.164 | 0.005 | -0.081 | 0.000 | -0.141 | 0.104 | 0.272 | 0.061 | -0.115 | 0.000 | 0.049 |
| ***Comamonadaceae*** | -0.375 | 0.618 | -0.941** | **0.530*** | 0.041 | -0.054 | 0.086 | 0.026 | -0.125 | -0.168 | -0.229 | -0.067 | 0.361 | 0.105 | 0.066 | 0.214 | -0.158 | -0.050 |
| ***Campylobacteraceae*** | 0.038 | -0.854 | 0.076 | 0.012 | **0.234** | -0.635 | -0.007 | -0.261 | 0.000 | 0.000 | 0.145 | -0.265 | -0.108 | -0.176 | 0.000 | 0.428 | -0.161 | 0.000 |
| ***Helicobacteraceae*** | -0.034 | 0.234 | 0.020 | 0.193 | -0.782** | **0.429**** | 0.030 | 0.214 | 0.607 | -0.451 | -0.149 | 0.152 | 0.000 | 0.018 | 0.000 | -0.552 | 0.057 | -0.722 |
| ***Acetobacteraceae*** | -0.606 | 0.347 | 0.608 | 0.183 | 0.393 | -0.246 | **0.216** | -0.094 | 0.061 | -0.149 | -0.210 | -0.017 | 0.157 | 0.105 | -0.051 | 0.135 | 0.055 | -0.059 |
| ***Bifidobacteriaceae*** | 0.000 | 0.053 | -0.506 | 0.449 | -0.382 | 0.605 | -0.787 | **0.233** | 0.121 | -0.050 | -0.206 | 0.138 | -0.003 | -0.040 | -0.330 | -0.181 | 0.213 | -0.245 |
| ***Rikenellaceae*** | 0.599 | 0.939 | 0.412 | -0.180 | 0.000 | 0.728** | -0.200 | 0.851 | **0.248** | -0.405 | -0.151 | 0.136 | -0.028 | -0.010 | 0.000 | -0.560 | -0.041 | 0.000 |
| ***Flavobacteriaceae*** | 0.603 | -0.759** | -0.034 | -0.586** | 0.000 | -0.742** | -0.175 | -0.445 | -0.258 | **0.400*** | 0.211 | -0.211 | -0.412 | -0.400 | 0.000 | 0.264 | 0.132 | 0.000 |
| ***Deinococcaceae*** | 0.886 | 0.000 | 0.000 | -0.732 | 0.452 | -0.157 | 0.431 | -0.519 | -0.386 | 0.705 | **0.280** | -0.023 | -0.329 | -0.237 | 0.126 | 0.029 | -0.010 | 0.339 |
| ***Acidaminococcaceae*** | 0.008 | 0.000 | -0.805 | -0.353 | -0.973 | 0.269 | -0.600 | 0.435 | 0.747 | -0.201 | -0.064 | **0.202** | 0.069 | 0.102 | -0.208 | 0.021 | 0.212 | -0.253 |
| ***Paenibacillaceae*** | -0.747** | 0.919 | -0.442 | 0.947** | -0.877 | 0.000 | -0.112 | 0.841 | 0.365 | -0.921 | -0.471 | -0.060 | **0.238** | 0.722 | 0.000 | 0.250 | -0.261 | 0.000 |
| ***Bacillaceae*** | -0.618 | 0.785 | 0.066 | 0.438 | -0.050 | 0.102 | 0.085 | 0.685 | -0.009 | -0.297 | -0.171 | 0.114 | 0.763** | **0.409*** | 0.050 | 0.191 | -0.280 | -0.260 |
| ***Aerococcaceae*** | -0.469 | -0.571 | -0.343 | 0.291 | 0.000 | 0.000 | 0.512 | -0.290 | 0.000 | 0.000 | 0.299 | -0.456 | 0.000 | -0.297 | **0.310** | 0.000 | -0.121 | 0.000 |
| ***Enterococcaceae*** | -0.519 | 0.166 | -0.799** | 0.421 | 0.753** | -0.545** | 0.246 | -0.340 | -0.911** | 0.680 | -0.353 | -0.047 | -0.358 | 0.055 | 0.000 | **0.473**** | -0.016 | 0.487 |
| ***Lactobacillaceae*** | 0.134 | -0.224 | 0.000 | -0.173 | 0.165 | -0.367 | -0.297 | 0.018 | -0.154 | 0.569 | 0.042 | 0.740 | -0.638 | -0.737** | 0.713 | 0.678 | **0.402** | 0.015 |
| ***Streptococcaceae*** | 0.079 | -0.707 | 0.007 | -0.446 | 0.000 | -0.805** | 0.253 | -0.612 | 0.000 | 0.000 | 0.386 | -0.521 | 0.000 | -0.372 | 0.000 | 0.478 | 0.342 | **0.360*** |


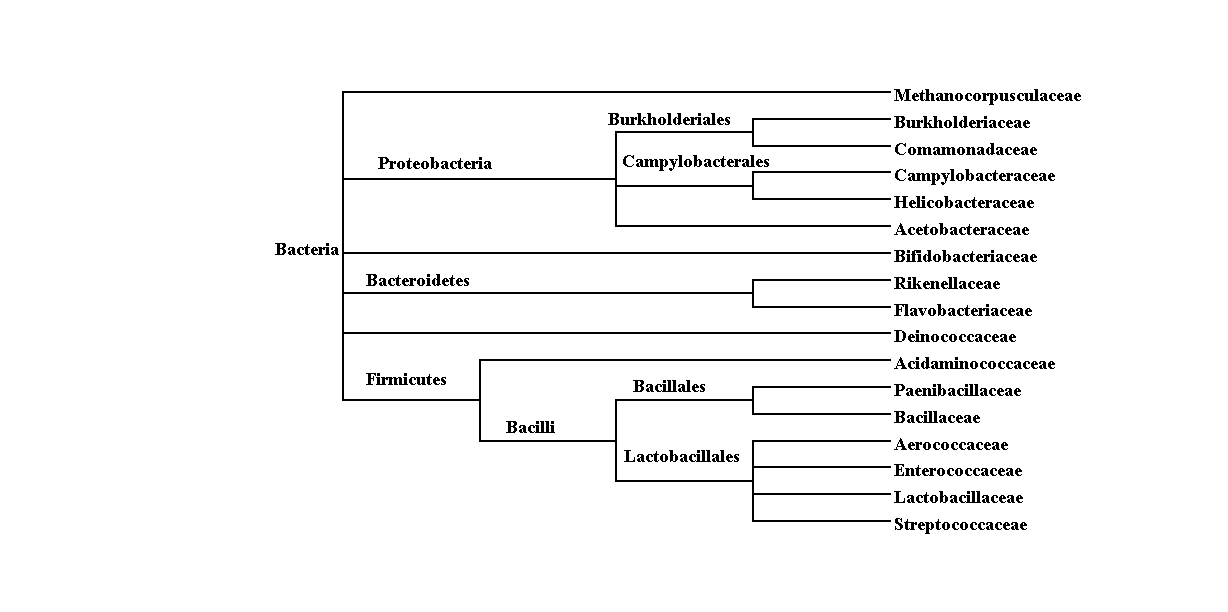

Supplement: Table S2 — Genetic and phenotype correlations for line LW. (DOCX) [file pone.0089862.s003.docx]
