# Supplementary material for: Body Weight Selection Affects Quantitative Genetic Correlated Responses in Gut Microbiota
Source: PLoS One. 2014 Mar 7;9(3):e89862. doi: 10.1371/journal.pone.0089862 (PMC3946484; doi:10.1371/journal.pone.0089862)
Supplement: Table S3 — Body weight and egg production of HW and LW lines of chicken. (DOCX) [file pone.0089862.s004.docx]

# Supplementary Table S3: Means and standard deviations of body weights at several ages for the populations from which the high(HW) and low(LW) weight line male and female samples were taken are shown below. Also shown is egg production for the females.

|  | **HW(g)** | | **LW(g)** | |
| --- | --- | --- | --- | --- |
| **age(days)** | **Male** | **Female** | **Male** | **Female** |
| **28** | **620+72(n= 75)** | **551+61(n= 78)** | **68+16(n=128)** | **56+16(n= 162)** |
| **56** | **1976+193(n=75)** | **1598+166(n=78)** | **174+46(n=128)** | **128+37(n= 162)** |
| **168** |  | **2740+180(n=72)** |  | **734+144(n= 80)** |
| **256** |  | **3313+281(n= 67)** |  | **1224+162(n= 78)** |
| **Hen-day ovulations (%)** | | **59+11(n= 71)** |  | **48+13(n= 61)** |
